# Optimization of a wheat small red bean double cropping system in South Korea

**DOI:** 10.1038/s41598-022-17681-3

**Published:** 2022-08-03

**Authors:** Jing Yang, Young-Bok Kim, Ki-Heung Hong, Seong-Tak Yoon

**Affiliations:** 1 Biology Department, Xinzhou Teachers University, Xinzhou, China; 2 Chungcheongnam-do Agricultural Research and Extension Services, Jonggyeong-ri, Sinam-myeon, Yesan-gun, 32418 Chungcheongnam-do Korea; 3 College of Bio-Resource Science, Dankook University, Cheonan, 31116 Chungcheonanam-do Korea

**Keywords:** Plant sciences, Plant breeding

## Abstract

Wheat (*Triticum aestivum* L.) and small red bean [*Vigna angularis* (Willd. Ohwi & Ohashi)] are the main ingredients of walnut-shaped “Hodugwaja”. An innovative wheat small red bean double cropping system was evaluated in a rice field in the Cheonan region (Korea) to determine its effect on land use. The effects of different nitrogen levels, sowing dates, and density on growth, yield, and quality of wheat and small red bean were also investigated using selected wheat (‘Keumgang’, ‘Sooan’, and ‘Goso’) and small red bean (‘Hongeon’, ‘Chungju’, and ‘Arari’) varieties. The effect of different fertilizer treatments [N1 (50%, 6.6 kg/10a), N2 (100%, 8.8 kg/10a), and N3 (200%, 13.2 kg/10a)] were investigated for wheat, while the effect of sowing date and density were investigated for the small red beans. Our findings revealed that the best variety, sowing date, and nitrogen level combination for wheat small red bean double cropping system in Cheonan area is ‘Goso’ sown on October 26, N3 nitrogen application, and ‘Chungju’ sown on July 10 with high ridge cultivation, at a density of 60 × 15 cm. This system was the most ideal yielding 521.6 kg/10a (1000 m^2^) and 275 kg/10a of ‘Goso’ and ‘Chungju’, respectively. This pioneering research provides a reliable cultivation plan and theoretical basis for implementing the double cropping system of wheat small red beans in central Korea. Undeniably, this study also provides a basis for future field experiments on wheat planting patterns and small red bean fertilization.

## Introduction

Double cropping refers to planting several crops in the same area and in the same crop year so that the same land is used to generate more than one crop per year. Several double cropping systems have been described and adopted globally due to their imminent benefits. Notably, double cropping could solve the current global food growth crisis. Some of the advantages of double cropping include (1) high single-year crop yield, thereby achieving effective crop production and reducing land consumption; (2) efficient land use through permanent protection of soil fertilization and excess nutrient loss^1^; (3) increased biodiversity^2,3^.

According to Graß et al.reported^4^, double cropping systems can achieve biomass yields similar to or higher than single cropping systems (depending on the type of variety and crop combination). Besides, several studies have shown that double cropping systems can also establish temporal diversity and increase land-use efficiency by increasing the annual biomass production in each season while protecting the environment^5,6^. However, the choice of crops in a double cropping system is influenced by the market demands.

Cheonan Walnut cookie, commonly known as “Cheonan Hodugwaja”, is a traditional specialty snack in the Cheonan region, Korea. Hodugwaja was first produced in 1934 by a couple (Gwigeum Jo and Boksun Sim) in Cheonan using traditional Korean confectionery^7^. It became popular in the 1970s and was sold at the Cheonan train station and on the trains using catering trolleys. Currently, it has an international market besides Korea.

Although wheat (*Triticum aestivum* L*.*) and small red bean [*Vigna angularis* (Willd. Ohwi & Ohashi)] are the main ingredients of the Cheonan Hodugwaja, Korea imports these cereals to meet the national deficit. The annual average wheat production in Korea was 2.82 million tons (MT) from 2007 to 2017, with wheat imports increasing from 3.45 MT in 2007 to 4.61 MT in 2017, mainly from the US^8^. The domestic annual average production and imports were 0.4 MT^9^ and 23.3 MT between 2006 and 2016, respectively. For two decades, efforts have been made to develop new varieties and foods of wheat and expand the wheat cultivation area in Korea^10,11^. In 2017, the small red bean planting area in Korea was 4,386 hectares, with a yield of 5,001 MT. Although the small red bean planting area is second only to soybean, the yield is lower than soybean. However, the small red bean has excellent adaptability to climate and soil, making it suitable for use in various planting systems^12^. Besides being used in staple foods like red bean porridge and red bean rice, small red bean serves as ingredients in rice cakes, bread, and candies^13^.

This study sought to determine whether a double cropping system of these two grains on a rice paddy field could increase their yields. Rice (*Oryza sativa* L.) is cultivated in mid-June after seedling transplantation and harvested in October^14^, while wheat is sowed in mid-September and harvested in mid-June in the central region of Korea^15^. Therefore, it is challenging to practice double cropping between wheat and rice due to the overlap of the rice planting period and wheat harvesting time (June)^15^. In the southern region of Korea, the wheat small red bean planting system is still difficult as the small red bean is usually sown from late May to mid-June, which is likely to conflict with the wheat harvest from early to mid-June. Therefore, it was necessary to discuss the effect of small red bean replacing rice and delaying the sowing date of small red bean on yield and growth.

In addition, the double cropping system for these two crops may be feasible due to global warming, which increases the temperature, thus affecting the crop cultivation system and season. The average temperature in Korea has risen by 1.5 °C over the past decade, with a higher overwintering temperature than the daily average minimum temperature of − 15 °C in January^16^. The double cropping system of wheat and small red beans can be achieved in Cheonan since the average temperature was − 7.6 °C in January between 2014 and 2017^17^, which is above the − 15 °C for wheat survival in winter^18^.

However, varieties and cultural practices should be investigated to maximize the yields of the two crops via the double cropping system in central Korea. Besides, South Korea has developed new red bean varieties, including ‘Seona’ (2014) and ‘Hongjin’ (2015), which have excellent agronomic traits such as high yield and lodging resistance. The standard planting density in Korea is 60 × 10^−15^ cm^19,20^. South Korea has also developed wheat cultivation techniques and high-quality wheat varieties^21^. Furthermore, in-depth research has been conducted on the quality and yield of wheat using varied amounts of fertilizers^22^.

Most of the agricultural land in Korea is paddy fields, which are widely used as alternative land for other food crops^23^. Our attempt to establish such a planting pattern in rice fields has very positive and enlightening significance for future agricultural production in Korea. There was no obstacle to the production of wheat in time, and the key factor affecting the yield was the amount of fertilizer applied. Because it was necessary to explore the sowing time of small red bean in double cropping system, we pay attention to the planting time and planting density. In order to explore the planting mode with the highest yield of the two crops in the double cropping system.

This research sought to determine the optimal varieties, cultivation methods, and fertilizer levels for establishing a double cropping system of wheat and small red beans in a rice paddy field in central Korea. The study majored on (1) selection of suitable varieties of wheat (‘Keumgang’, ‘Sooan’, and ‘Goso’) and small red bean (‘Hongeon’, ‘Chungju’, and ‘Arari’), (2) three nitrogen treatments (6.6, 8.8, and 13.2 kg/10a), and (3) three sowing densities (60 × 15, × 20, and × 25 cm (ridge × spacing) sowed on (July 1, 10, and 20) for small red bean after harvesting wheat.

## Materials and methods

### Experimental plot and soil analysis

Experiments were conducted between 2015 and 2017 at Gwangduck-Myon, Dongnam-gu, Cheonan-si, and Chungcheongnam-do (36° 43′ 08.9" N 127° 05′ 28.6" E), using sandy loam soil (the Gocheon soil series). Soil analysis was conducted following the Korea Rural Development Agency method^24^. Soil samples were collected from topsoil (25 cm deep) at the three locations per plot, air-dried, and sieved through a 2 mm sieve before analysis.

The soil samples were mixed with distilled water at a 1:5 ratio (weight: volume), and a pH meter (Orion Star A215 pH/conductivity benchtop multi-parameter meter (Thermo Scientific—Thermo Fischer Scientific, Waltham, MA, USA) was used to analyze the soil pH after an hour. The Tyurin method^25^ was used to assess the soil organic matter (OM). The slurry was diluted with a 0.4 N K_2_Cr_2_O_7_ solution, and the Lancaster method^26^ was used for extraction. A bismuth carbonate (UV–Vis) spectrophotometer (Agilent Technologies, Palo Alto, CA, USA) was used to measure the optical density.

An atomic absorption spectrophotometer (5100 ICP-OES, Agilent Technologies, Palo Alto, CA, USA) was used to analyze the exchangeable cations from the filtrate [5 ml of 50 ml of 1 N NH_4_OAc (pH 7.0) soil mixed with slurry and stirred for 30 min]. The conductivity benchtop multi-parameter meter (Orion Star A215, Thermo Scientific, MA, USA) was used to measure the conductivity of the soil solution, and the conductivity values were converted at the reference temperature (25 °C).

### Wheat and small red bean cultivation and management

#### Wheat variety evaluation and nitrogen treatments

We evaluated three improved wheat varieties, ‘Keumgang’, ‘Sooan’, and ‘Goso’ from the National Institute of Crop science, Rural Development Administration (RDA), and Jeon-ju, Korea, respectively. The seeds were sterilized using a water-soluble Thiram (26.5%, 30 Agro, Seoul, Korea) and powdered carboxymethyl chitosan (Carboxin-37.5, thiram-37.5, Dongbang Agro, Seoul, Korea) a day before sowing.

The experiment was a split-plot design with different nitrogen fertilizer treatments and varieties, with three replications. Seeds were sown on October 26 annually at a seeding volume of 180 kg per hectare from 2015 to 2017. Fertilizer application was in line with the recommendations of the Rural Development Administration^27^. A standard amount of Nitrogen-Phosphorus-Potassium (88–80–37 kg per ha) is used as the base treatment before sowing in the central and northern regions. In this experiment, a compound fertilizer (21 N-17P-17 K) was used as the base fertilizer and urea (46 N-0P-0 K) as the topdressing fertilizer. The nitrogen fertilizer was used as basic fertilizer and topdressing fertilizer, while phosphorus and potassium were used as basic fertilizers. During sowing, 210 kg per ha of compound fertilizer was used. The 100% fertilizer was applied on standard plot (N1), 50% on the shortage plot (N2), and 200% on the above-standard plot (N3) based on standard urea (46 N-0P-0 K), translating to 96 kg, 48 kg, and 192 kg per ha, respectively. Therefore, the nitrogen content per ha was 66 kg in plot N1, 88 kg in plot N2, and 132 kg in plot N3. Other cultural practices such as water, pest control, and weed control followed the standards of the Rural Development Administration^28^.

#### Evaluation of small red bean varieties and cultural practices

Three small red beans varieties, ‘Hongeon’, ‘Chungju’, and ‘Arari’ from the Rural Development Administration were assessed. The seeds were sterilized using a water-soluble seed fungicide (Thiram-26.5%, 30 Agro, Seoul, Korea) a day before sowing.

The ridge (25 cm high) was covered using a black polyethylene film after applying the base fertilizer. Seeds were sown on July 1st, 10th, and 20th at ridge distances of 60 cm. Besides, holes were punched at 15, 20, and 30 cm apart, setting sowing densities of each variety at 60 cm (between ridges) × 15 cm (between plants), 60 cm × 20 cm, and 60 cm × 30 cm.

The experiment was a split-plot design, with the sowing date, variety, and sowing density as the level one, two, and three treatments, respectively. The experimental block was 48 m^2^, with each block having four ridges (20 m long).

Three seeds were sown per hole, and seedlings were thinned after emergence, leaving two seedlings. In our preliminary study, having two seedlings per hole yielded high germination rates (90%) and was adopted in this study. In the central and northern regions, all small red bean fertilizers are standard nitrogen, phosphorus, and potassium (4.0 kg, 6.0 kg, and 6.0 kg per 10a (N-P_2_O_5_-K_2_O), respectively) as recommended by the RDA standards (RDA, 2005). In this study, 50 kg compound fertilizer (8 N-14P-12 K) was used per 10a as base fertilizer. Other field management measures, such as water, pest control, and weed control, were implemented following the standards of the Rural Development Administration^29^.

### Data collection of wheat growth and yield

The heading date was recorded when 40% of plants (per variety) per replication reached the heading based on the Agricultural Science and Technology Research Survey and Analysis Standard of the Rural Development Agency^30^. The culm and spike lengths were also recorded on the heading date. The yield per 10a (1000 m^2^) was calculated based on yield components including, the number of ears, the thousand-grain weight, and the number of grains per ear. The unit replication area was recorded following the standards of the Rural Development Agency^28^.

The number of spikes was recorded from 10 plants from each replication block, 25 days after heading. In addition, the number of grains per spike was recorded for three spikes per replication and replicated three times.

The leaf color was also measured after nitrogen fertilizer treatment. A Chlorophyll Meter (SPAD 502, Minolta, Japan) was used to detect chlorophyll 20 days after heading from the mid-position of the leaf blade of the uppermost leaf collected between 10:00 and 11:00 am (20 blades). The leaves were dried and ground using a mortar and pestle before determining their nitrogen content in an elemental analyzer (CN elementary analyzer, Vario Max, Germany). Grains were harvested when moisture content reached 20%. A grain moisture meter (GMK-303RS, G-won, Korea) was used to measure the moisture content.

### Data collection of small red bean growth and yield characteristics

Flowering date, stem length, and the number of pods were recorded from 10 plants per replication in triplicates following the Agricultural Science and Technology Analysis Standard^30^. According to the yield components per square meter, the yield per10a (1000 m^2^) was calculated based on yield components including, the number of plants, the number of pods per plant, the number of seeds per pod, and the weight of 1000 seeds.

The flowering date was recorded when 40% of the plants in a block bloomed. The plant height was measured from the ground to the shoot spike tip, and the number of pods per plant was recorded, excluding empty pods. Ten plants in the second and third rows were selected for the survey following the Agricultural Science and Technology Survey and Analysis Standards^30^. The small red bean was harvested once the tip of the pod turned yellow, and moisture content was reduced to 14%. Besides, the weight of 100 grains was recorded when the moisture content was less than 13%.

### Climate conditions during wheat small red bean double cropping cultivation

The global average temperature was highest in 2016, with Korea experiencing the hottest summer^17^. Meteorological data from 1981 to 2010 and experiments conducted between 2015 and 2017 in the Cheonan Meteorological Center (2017) are shown in Table 1. Global warming affects the flowering of many crops and global ecosystems^31^.

**Table 1:** Meteorological data during wheat and small red bean cultivation periods in Cheonan area.

Period	Wheat culture period (2015–17)										Small red bean culture period (2015–17)				
	Oct^z^	Nov	Dec	Jan	Feb	Mar	Apr	May	Jun^y^	Oct–Jun	Jul^w^	Aug	Sep	Oct^x^	Jul–Oct
**Daily minimum temperature (**°C**)**															
Normal year	4.0	0.8	− 4.9	− 7.9	− 5.6	− 1.0	4.7	11.2	16.1	1.9	20.9	21.0	15.0	8.5	16.4
2015–2017	4.6	1.4	− 4.4	− 6.7	− 4.7	− 0.9	6.5	10.9	16.1	2.5	21.7	21.1	15.1	10.1	17.0
**Daily maximum temperature (**°C**)**															
Normal year	17.3	12.5	5.4	2.5	5.3	11.2	18.6	23.7	27.1	13.7	29.4	30.1	26.0	21.6	26.8
2015–2017	16.1	12.2	5.5	3.5	5.8	12.9	20.0	25.3	28.1	14.4	29.5	30.5	26.4	21.9	27.1
**Daily average temperature (**°C**)**															
Normal year	10.2	6.2	− 0.1	− 2.9	− 0.3	4.8	11.4	17.2	21.2	7.5	24.7	25.1	20.0	14.5	21.1
2015–2017	10.0	6.6	0.4	− 1.6	0.5	5.9	13.1	18.1	21.8	8.3	25.2	25.2	20.3	15.5	21.6
**Accumulative temperature (**°C**)**															
Normal year	61	186	17	0.0	13	150	343	533	531	1,835	766	777	601	305	2,448
2015–2017	60	199	31	1.7	34	183	394	560	546	2,008	780	783	609	326	2,497
**Precipitation (mm)**															
Normal year	8	50	28	23	29	46	61	88	99	430	268	293	157	41	758
2015–2017	23	97	69	28	71	26	172	95	63	642	766	295	88	95	1,243
**Sunshine hour (h)**															
Normal year	6.8	5.5	5.2	5.6	6.5	7.0	7.8	8.0	7.7	6.7	6.0	6.7	6.7	7.1	6.6
2015–2017	5.8	4.8	5.4	5.1	6.1	7.7	6.9	8.7	7.5	6.4	4.5	6.6	6.6	5.9	5.9

^z^Oct. 26–Oct. 31, ^y^June 1–June 25. The small red bean was also harvested when the tip of the pod turned to yellow.

^x^Oct. 1–Oct. 21, ^w^July. 16. 2017 Daily precipitation: 232.7 mm/day.

Meteorological data between 1981 and 2010 (Normal year) and from 2015 to 2017 (experiment period) were obtained from the Cheonan Meteorological Center (Table 1). The minimum, maximum, and average temperatures were higher from 2015 to 2017 than from 1981 to 2010 throughout the year. Daily minimum temperatures were higher from 2015 to 2017 than in 1981–2010 (0.5–1.2 °C from November to May and not more than 0.2 °C from July to September). The precipitation in the experiment period was higher than the Normal year throughout the year, especially from October to December when wheat emerges and tillers. However, the sunshine duration in the experiment period was slightly lower than the Normal year throughout the year.

Since arable land has become limited due to the rapid urbanization in the late twentieth century, a double cropping system could achieve efficient land use. In Cheonan, wheat is planted in October and harvested in June the following year^28^ due to the high temperatures in the region during winter (Table 1). Meanwhile, the small red bean is sown in June and harvested in October of the same year^32^. Therefore, the double cropping system of wheat and small red beans could succeed in central Korea, especially utilizing the rice field during reduced rice consumption.

### Soil conditions during wheat small red bean double cropping cultivation

The chemical properties of the soil samples were within the optimum ranges established by Standards for fertilization of soil^33^ except for the exchangeable potassium (K^+^) and pH level (Table 2). For instance, Organic matter was 22 g/kg, phosphorous was 225 g/kg, and electric conductivity was 1.0 dS/m. However, the exchangeable K^+^ level was 0.66 cmol/kg, slightly higher than the acceptable range, while the pH value was slightly lower (6.0) than the optimal pH between 6.5 and 7.0 (Table 2). Therefore, the soil used was suitable for wheat and small red bean cultivation^30^.

**Table 2:** Chemical characteristics of the soil.

	pH (1:5)	OM (g/kg)	P_2_O_5_ (mg/kg)	Ex. Cation (cmol../kg)			EC (dS/m)
				K^+^	Ca^++^	Mg^++^	
Field	6.0	22	225	0.66	5.9	1.9	1.0
Proper value^z^	6.5–7.0	20–30	150–250	0.45–0.55	6.0–7.0	2.0–2.5	0.0–2.0

^z^Proper value: Standards for soil fertilization, National Institute of Agricultural Science and Technology, Rural Development Administration. RDA (2010).

### Statistical analysis

SAS software (Version 9.2, Statistical Analysis System, SAS Institute, Cary, NC, USA) was used for data analysis. ANOVA (Analysis of Variance) was used to compare means via the least significant difference (LSD) at 1% and 5% significance levels.

### Experimental statement

We confirm that the seeds in this manuscript have been authorized and licensed.All experimental methods are carried out according to the relevant guidelines of Korea Academy of Agricultural Sciences. All data generated or analysed during this study are included in this published article.

## Results and discussion

### Wheat growth

#### Late October to mid-June

**Figure 1:**
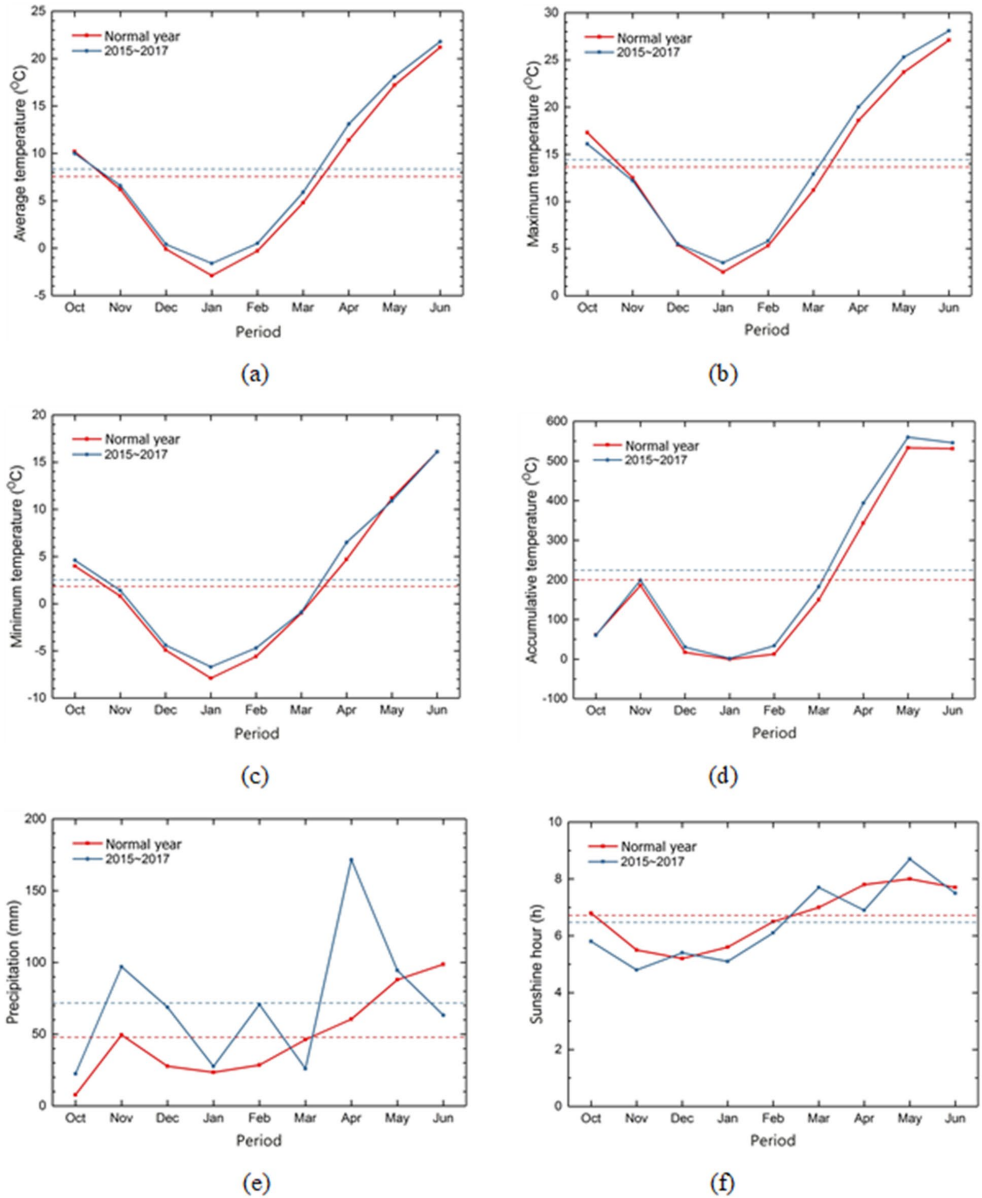
Meteorological environments during wheat cultivation periods in Cheonan area; (**a**) daily average temperature (°C), (**b**) daily maximum temperature (°C), (**c**) daily minimum temperature (°C), (**d**) cumulative temperature (°C), (**e**) precipitation (mm), and (**f**) sunshine hours (h).

The average, maximum and minimum temperatures increased by 0.8 °C, 0.7 °C, and 0.6 °C, respectively, during the wheat cultivation period between 2015 and 2017, compared with the previous years (1981–2010). Similarly, the IPCC report (3rd and 5th) pointed out that global warming increases the average earth temperature^34,35^. The effective accumulated temperature of the days when the daily average temperature is above 0 °C increased by 173 °C during the wheat cultivation period in 2015–2017, higher than in previous years (1981–2010). The monthly average temperature decreased only in October during the sowing period and increased from November (Table 1) (Fig. 1).

The suitable period for wheat sowing in South Korea is between mid and late October based on the standard agricultural recommendations^28^. Wheat was sown on October 26 in the Cheonan area, considering the harvest time of the two crops. Furthermore, the average monthly meteorological factors and changing trends were investigated until June 25, the harvest period of the second year.

In addition, in autumn wheat, the average minimum temperature in January reached -15°C to safely over-winter for wheat^16^, which is lower than − 5.0 and − 7.9 °C in mid-to-late October^28^. The low temperature was due to increased precipitation (over 212 mm) and reduced sunshine duration (less than 0.3 h) in the experiment period. The precipitation between October and December is critical for ensuring the increased number of wheat grains per year (Fig. 1e, Table 1). The precipitation increased by 15, 48, and 41 mm, respectively, between 2015 and 2017, higher than in previous years. However, the precipitation during the harvest period (June) decreased by 1.6 times (35 mm). The average temperature increased from late October to November over the experiment period. Late October was a suitable planting period due to the 23 mm precipitation. The average temperature increased in June during the harvest period, while the precipitation gradually decreased, indicating that wheat can be cultivated over the winter in the Cheonan area (Fig. 1, Table 1).

Heading of three wheat varieties occurred between 24 and 27 April (Table 3), coinciding with the nationwide heading date^36^. However, since the daily average temperature in mid-February 2017 in the central region where Cheonan is located was 0.4 °C higher than in 2016, the heading dates of three varieties in 2017 were all two days earlier than in 2016. Therefore, the difference is related to the high temperatures^37^. The precipitation and sunshine duration were lower (79.7 mm and 6.7 h, respectively) in 2017 than in 2016 due to the recent global warming in South Korea^38^.

**Table 3:** Meteorological environments and heading date of wheat in Cheonan area.

Variety	Heading date		Average Temperature (°C)^z^		Precipitation (mm)^y^		Sunshine (h)^x^	
	2016	2017	2016	2017	2016	2017	2016	2017
Keumgang	4/27	4/25	7.8	7.4	149.3	79.7	6.7	7.7
Sooan	4/26	4/24						
Goso	4/27	4/25						

^zyx^Every year from February 15 to April 25.

### The effect of additional nitrogen fertilization on SPAD and plant N content

Photosynthesis peaked in mid-April and gradually decreased 21 days after heading, before significantly declining during the wheat growth period^28^. In this study, the effects of different fertilization treatments on the SPAD value and leaf nitrogen content 20 days after heading were investigated. The SPAD was associated with nitrogen content in wheat leaves, consistent with previous results in rice (*Oryza sativa L.*)^39,40^.

The average SPAD values of ‘Keumgang’, ‘Sooan’ and ‘Goso’ were 54.0, 53.9, and 48.3, respectively, after different nitrogen fertilizer treatments. There SPAD values between ‘Keumgang’ and ‘Sooan’ were not different, while ‘Goso’ had the lowest average SPAD value. However, the average yield was significantly different among the varieties, with ‘Goso’ having the highest (496.7 kg/10a) and ‘Keumgang’ the lowest (452.4 kg/10a). ‘Goso’ also had the lowest average leaf nitrogen concentration (3.69%), while ‘Sooan’ had the highest average (4.33%) (Table 4).

**Table 4:** SPAD value and N concentration of wheat at Kwangdeok in Cheonan area.

Variety	Nitrogen level (kg/10a)	SPAD value^z^	N cont. (%) ^y^	Yield (kg/10a)
Keumgang	6.6 (N1)^x^	51.7de	3.98bc	434.0c
	8.8 (N2)	54.4abc	4.32ab	439.6bc
	13.2 (N3)	55.9a	4.41a	483.6abc
	Mean	54.0	4.24	452.4
Sooan	6.6 (N1)	52.6cde	4.11abc	450.6abc
	8.8 (N2)	53.6bcd	4.33ab	492.3abc
	13.2 (N3)	55.4ab	4.51a	519.0ab
	Mean	53.9	4.33	487.3
Goso	6.6 (N1)	47.1f	3.39d	461.0abc
	8.8 (N2)	48.4f	3.74cd	507.6abc
	13.2 (N3)	51.0e	3.93bc	521.6a
	Mean	48.3	3.69	496.7
Varieties		**	**	*
Nitrogen level		**	**	**
Varieties*nitrogen level		NS	NS	NS
Block		*	**	*
LSD		1.885	0.378	70.3

*NS* non-significant.

Level of significance; *, **: significant at P < 0.05, 0.01.

^zy^Data collected on the 20th day at or after heading.

^x^N1(50%, 6.6 kg/10a), N2(100%, 8.8 kg/10a), N3(200%, 13.2 kg/10a).

Therefore, Nitrogen fertilizer levels significantly affect the yield. Kim et al.indicated that the leaf color concentration should be determined before wheat cultivation for proper fertilization^39^. The leaf SPAD in each variety was highest under the N3 treatment but significantly lower under the N1 treatment relative to N3. ‘Keumgang’ leaves had the highest nitrogen concentration (4.41%). The yield per 10a was also highest under N3 treatment and lowest under N1 treatment, with ‘Goso’ having the highest yield (521.6 kg/10a) (Table 4).

### Effect of additional nitrogen fertilizers on the growth characteristics of wheat

The Rural Development Administration recommends 9.4 kg of nitrogen fertilizer per 10a as a food quality standard^27^ for wheat in South Korea. However, most farmers do exceed the recommended quantity. In this experiment, the growth and yield of wheat were analyzed at different nitrogen fertilizer levels.

In the central region of South Korea, nitrogen is traditionally applied once before the elongation of wheat internode to avoid delay in wheat maturity. Therefore, three different nitrogen fertilizer treatments were used according to the existing cultivation method. The wheat growth characteristics, such as culm and spike lengths, were recorded during the nitrogen experiments in a two-year wheat small red bean double cropping system in the Cheonan area (Table 5).

**Table 5:** Growth characteristics of wheat according to levels of nitrogen fertilization at Kwangdeok in Cheonan area.

Variety	Nitrogen level (kg/10a)	Culm length (cm)	Spike length (cm)
Keumgang	6.6 (N1)^x^	68.1b	7.3c
	8.8 (N2)	69.4b	7.6bc
	13.2 (N3)	71.0b	7.6bc
	Mean	69.5	7.5
Sooan	6.6 (N1)	81.8a	7.3c
	8.8 (N2)	79.4a	7.5bc
	13.2 (N3)	80.3a	7.4c
	Mean	80.5	7.4
Goso	6.6 (N1)	68.7b	8.0ab
	8.8 (N2)	67.8b	8.4a
	13.2 (N3)	69.0b	8.5a
	Mean	68.5	8.3
Varieties		**	**
Nitrogen level		NS	NS
Varieties*nitrogen level		NS	NS
Block		**	NS
LSD		7.99	0.525

*NS* non-significant.

ANOVA’s test; **, significant at P < 0.01.

^z^N1(50%, 6.6 kg/10a), N2(100%, 8.8 kg/10a), N3(200%, 13.2 kg/10a).

The culm length is directly related to stem mechanical properties and lodging resistance^40^. The average stem length was 69.5, 80.5, and 68.5 cm in ‘Keumgang’, ‘Sooan’, and ‘Goso’, respectively (Table 5).

The average spike lengths of ‘Keumgang’, ‘Sooan’ and ‘Goso’ were 7.5, 7.4, and 8.3 cm, respectively, and did not differ significantly among the nitrogen fertilization levels (N1, N2, N3) (Table 5). Kim et al. had previously reported similar results^11^, where spike length was not directly associated with nitrogen levels, while culm length increased with higher nitrogen fertilization levels. Although the culm and spike lengths were significantly different among the three varieties in our study, the difference was not significant among the three treatments. The differences could be due to climate and soil conditions during internode elongation (Table 5).

The temperature and sunshine duration comparison between Cheonan and southern regions (Gwangju, Jeon-ju) from March to April during the internode growth period of wheat is shown in Table 6. The average and maximum temperatures were lower by 1.2–1.8 °C and 0.8–1.4 °C, respectively, in the Cheonan area compared with the southern area. However, the sunshine duration was similar in both regions.

**Table 6:** Meteorological data during internode elongation growth stage of wheat from 2015 to 2017 in Cheonan, Jeon-ju and Gwangju area.

Period	Mar	Apr	mean
**Daily average temperature (**°C**)**			
Cheonan	5.9	13,1	9.5
Jeon-ju	7.1	14.2	10.7
Gwangju	7.9	14.8	11.3
**Daily maximum temperature (**°C**)**			
Cheonan	12.9	20.0	16.5
Jeon-ju	13.7	20.8	17.3
Gwangju	14.4	21.4	17.9
**Sunshine hour (h)**			
Cheonan	7.7	6.9	7.3
Jeon-ju	7.5	6.9	7.2
Gwangju	7.5	7.1	7.3

The internode growth stage was consistent among reports related to meteorological factors, such as the highest temperature, average temperature, and sunshine duration between March and April^41^.

### The effect of nitrogen fertilizers on wheat yield

Cook and Baten reported that nitrogen fertilization significantly increases the number of wheat ears^42^. Nitrogen fertilizer significantly increases the number of ears and yield than 1000-grain weight in barley, thus increasing the yield per unit area^43^. Besides the number of ears, other components are also associated with increased yield^44,45^. Kim et al. also reported that the number of ears and 1000 grain weight substantially affect barley yield^46^. Moreover, increasing fertilization was more effective than increasing sowing seeds in wheat^47,48^. However, the high yields of some high-yielding wheat regions are due to genotype improvement, mechanization and the application of large amounts of nitrogen fertilizer and other pesticides. This intensification level depends largely on fossil fuels and may not be sustainable^49^.

The yield-related characteristics of wheat, based on the varieties and nitrogen fertilization levels, are shown in Table 7. The average number of grains per ear of the three varieties ‘Keumgang’, ‘Sooan’ and ‘Goso’ were 38.1, 37.2, and 42.3, respectively.

**Table 7:** Yield components of wheat according to levels of nitrogen fertilization at Kwangdeok in the Cheonan area.

Variety	Nitrogen level (kg/10a)	Number of grains/ spike	Number of spikes (m^2^)	1000 grains Wt. (g)	Yield (kg/10a)
Keumgang	6.6 (N1)^x^	37.4de	534.8bc	39.8a	434.0c
	8.8 (N2)	37.6de	607.8ab	40.4a	439.6bc
	13.2 (N3)	39.4cd	656.5a	40.7a	483.6abc
	Mean	38.1	599.7	40.3	452.4
Sooan	6.6 (N1)	35.7e	609.6ab	39.5a	450.6abc
	8.8 (N2)	37.6de	649.2a	40.9a	492.3abc
	13.2 (N3)	38.4cd	681.3a	39.7a	519.0ab
	Mean	37.2	646.7	40.0	487.3
Goso	6.6 (N1)	40.7bc	512.0c	38.9a	461.0abc
	8.8 (N2)	42.6ab	672.8a	38.8a	507.6abc
	13.2 (N3)	43.7a	696.1a	38.9a	521.6a
	Mean	42.3	627.0	38.9	496.7
Varieties		**	NS	NS	*
Nitrogen level		**	**	NS	**
Varieties*nitrogen level		NS	NS	NS	NS
Block		*	NS	*	*
LSD		2.414	88.847	3.162	70.38

*NS* non-significant.

ANOVA’s test; *, **Significant at P < 0.05, 0.01.

^z^N1(50%, 6.6 kg/10a), N2(100%, 8.8 kg/10a), N3(200%, 13.2 kg/10a).

The number of grains per panicle was highest under N3 treatment, contrary to Kim et al.^11^. In their study, the characteristics of the variety significantly affected the number of grains per ear than the fertilizer type and levels.

The average number of ears per m^2^ for ‘Keumgang’, ‘Sooan’ and ‘Goso’ were 599.7, 646.7, and 627.0, respectively, showing significant differences among the varieties. However, the number of ears per m^2^ was highest under N3 treatment. Ullah et al. reported similar results^50^, indicating the influence of nitrogen fertilizer levels.

Furthermore, the average 1000 grain weight of ‘Keumgang’, ‘Sooan’, and ‘Goso’ was 40.3, 40.0, and 38.9 g, respectively, exhibiting no significant difference among the varieties. This finding could be due to the influence of the unique characteristics of varieties, consistent with Kim et al.^51^. Hobbs et al.reported that nitrogen treatment significantly increases the number of ears per unit area and grains per ear than the 1000-grain weight^52^, indicating that the characteristics of the crop variety affect the weight of 1000 grains.

The average yields of ‘Keumgang’, Sooan’ and ‘Goso’ were 452.4, 487.3, and 496.7 kg per 10a, respectively. Besides, the yields were significantly different at the three nitrogen fertilizer levels (Table 7), with ‘Goso’ having the highest yield (521.6 kg/10a) under N3 treatment. The yields increased with higher nitrogen fertilizer application. Therefore, nitrogen fertilizer substantially influences wheat yield^42,53,54^. Ayoub et al.reported that final wheat yield increases with higher nitrogen fertilizer levels, similar to this study^55^.

### Growth characteristics of small red bean

#### Optimum sowing time of small red bean as a second crop after wheat

##### Early July to mid-October

**Figure 2:**
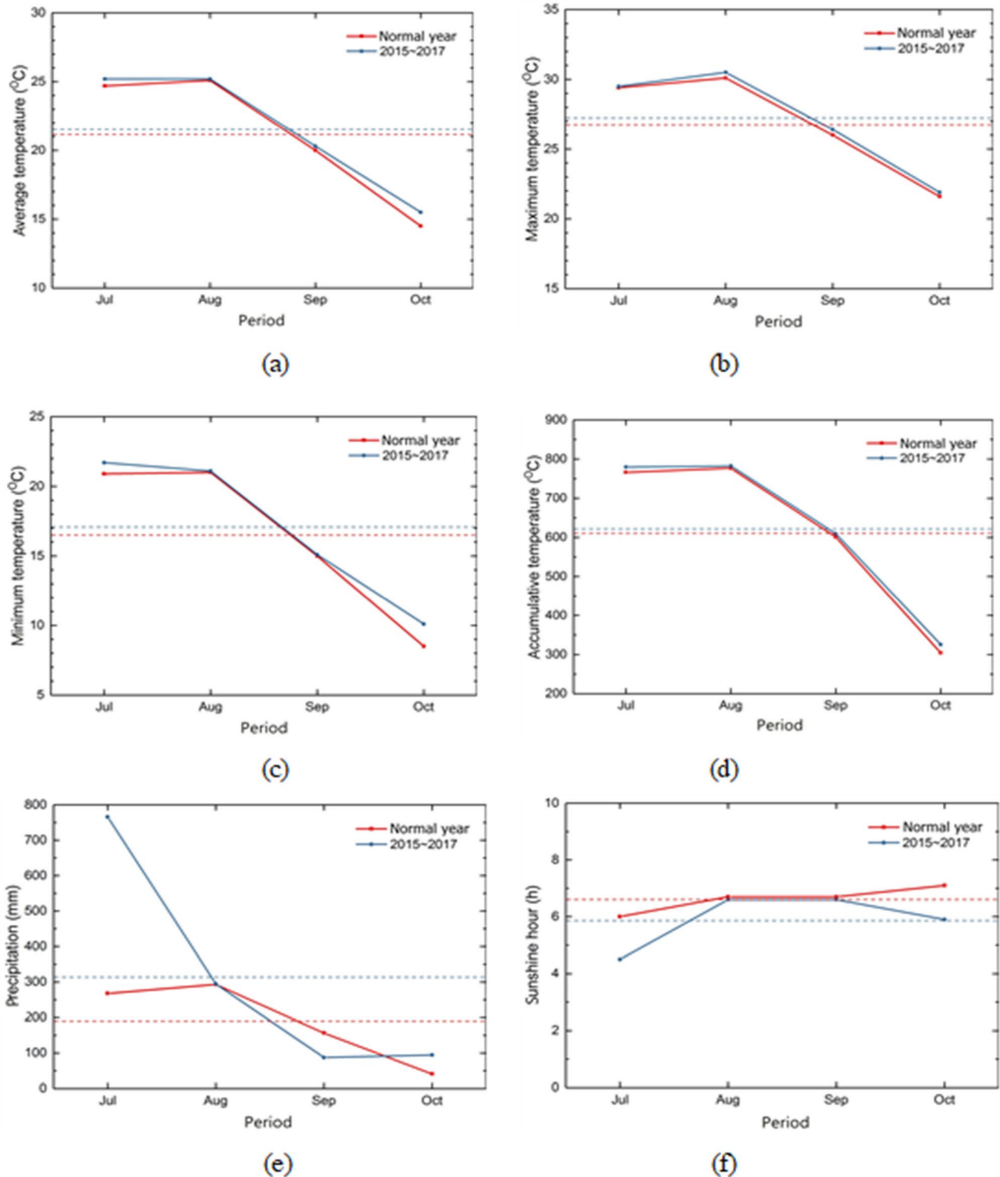
Meteorological data during small red bean cultivation periods in Cheonan area; (**a**) daily average temperature (°C), (**b**) daily maximum temperature (°C), (**c**) daily minimum temperature (°C), (**d**) accumulative temperature (°C), (**e**) precipitation (mm), and (**f**) sunshine hour (h).

The average, maximum and minimum temperatures increased by 0.5 °C, 0.3 °C, and 0.6 °C, respectively, during the small red bean breeding period between 2015 and 2017, higher than the normal year (1981–2010). The temperature increase in winter was greater than that in summer during the red bean growth period compared with the wheat growth period, similar to the previous research result^56^. The precipitation was 485 mm higher in 2015–2017 than in the normal year. This could be attributed to the torrential rainfall of 233 mm in the Cheonan area on July 16, 2017. However, the overall precipitation and the sunshine duration increased in the experiment period. In the past three years, the sunshine duration has decreased by 0.7 h compared with the normal year (Table 1) (Fig. 2).

Rural Development Administration^29^ and Kim et al. reported that the optimum sowing time for small red beans is between mid-June and mid-July in central Korea in single-season sowing^57^. However, the wheat has to be harvested after mid-June if small red beans are planted after wheat. Therefore, it is necessary to explore the possibility of planting in July.

Besides, delayed harvesting affects the small red bean varieties in Korea, including the ‘Chungju’, since over 90% of the small red bean have an intermediate growth type^58^. However, the recently developed varieties, such as ‘Hongeon’ and ‘Arari’, have a determinate and semi-determinate growth type^59^. Therefore, the sowing period can be extended based on the chosen variety.

The number of days from sowing to flowering in ‘Chungju’ was 49 days, 47 days, and 46 days, on July 1, July 10, and July 20, respectively. For ‘Arari’, it took 47 days, 46 days, and 46 days, respectively (Table 8). ‘Hongeon’ flowered earliest (33 to 34 days) among the varieties used in this study. Furthermore, our findings revealed that the unique characteristics of the varieties influenced the number of days to the flowering stage, consistent with the previous studies^60^.

**Table 8:** The cumulative temperature during the growing period of small red beans from 2015 to 2017 in the Cheonan area.

Variety	Sowing date (mm/dd)	Flowering date (mm/dd) and no. of days to from sowing	Cumulative temperature (°C)	Average temperature^y^ (°C)
Chungju	7/1	8/18 (49)	1257.1	25.7
	7/10	8/25 (47)	1222.0	26.0
	7/20	9/3 (46)	1168.3	25.4
	Mean	47.3	1215.8	25.7
Hongeon	7/1	8/2 (33)	833.9	25.3
	7/10	8/11 (33)	863.2	26.2
	7/20	8/22 (34)	899.3	26.5
	Mean	33.3	865.5	26.0
Arari	7/1	8/16 (47)	1205.2	25.7
	7/10	8/24 (46)	1197.8	26.0
	7/20	9/3 (46)	1168.3	25.4
	Mean	46.3	1190.4	25.7

^z^Days from seeding to flowering.

^y^Cumulative temperature ÷ Days from seeding to Flowering.

The growth temperature was suitable up to the flowering period when the three varieties were sown between July 1 and July 20 (Table 8). Besides, the number of days to flowering was also not significantly different between July and June sowing.

### Climate during small red bean growing period (early July to mid-October)

The average monthly meteorological elements from July 1, the first sowing date of small red bean grown after harvesting wheat, to October 21, the final harvesting date, are shown in Table 7.

The average, maximum, and minimum temperatures increased by 0.5 °C, 0.3 °C, and 0.6 °C, respectively, in the experiment period (2015–2017), higher than in the normal year (1981–2010). The temperature increase in winter was higher than in summer during the experiment period, consistent with a previous Korean study^56^. The precipitation was 484.4 mm in the experiment period was higher than in the normal year (1981–2010), with 232.7 mm of rainfall per day on the Cheonan area on July 16, 2017. However, overall precipitation increased between 2015 and 2017 except in September (2016). Sunshine duration also reduced by 0.7 h in the last three years compared with the normal year (Fig. 2, Table 1).

In the entire small red bean growth period, the cumulative temperature has been reported to be good for flowering and fruiting at 1000 °C or higher^61,62^. For the July 1, July 10, and July 20 sowing dates, ‘Chungju’ had cumulative temperatures from 1168.3 to 1257.1 °C, with an average of 47 days to flowering date (Table 8). However, ‘Arari’ had an average of 46 days. In both varieties, the cumulative temperature decreased with a delay of the sowing date.

‘Hongeon’ had an average of 33 days from sowing to flowering date for the three sowing dates of July 1, July 10, and July 20, with cumulative temperatures of 833.9 °C, 863.2 °C, and 899.3 °C, respectively. The cumulative temperature required for ‘Hongeon’ growth is also 1000 °C or higher. Therefore, the cumulative temperature does not influence growth and harvest when sowing before July 20 except for the ‘Hongeon’ in Cheonan.

### Effect of sowing date and density on small red bean yield

Several studies on yield characteristics based on sowing date have been reported on soybeans. Cha and Lee indicated that culm length increases in the dense planting regardless of the sowing time of the soybean when planted after harvesting barley^63^. Furthermore, the number of branches, pods per plant, and seeds per pod increases in the sparse planting plot.

Park et al. showed that the stem length increases with an increased number of plants per hill due to plant competition^64^. However, the stem length does not elongate enough to induce lodging since the cultivation period is short.

Besides, Rho et al. reported 100-seed weight, number of pods per plant, and number of seeds per pod as the yield characteristics of small red beans^12^. However, stem length and flowering date are indirect factors^65^.

The growth and yield characteristics of the small red bean based on the sowing date and density are shown in Tables 9 and 10. The analysis was conducted for three years (2015–2017) in the wheat small red bean double cropping system in the Cheonan area, the central area of South Korea.

**Table 9:** Growth characteristics of small red bean according to seeding date and seed spacing.

Variety	Sowing date (mm/dd)	Sowing density (cm)	Flowering date (mm/dd)	Stem length (cm)	No. of pods per plant
Chungju	7/1	60 × 15	8/18 (49)^z^	66.0a	31.0def
		60 × 20		65.3ab	37.3bc
		60 × 25		63.7abc	46.1a
	7/10	60 × 15	8/25 (47)	65.1ab	29.8ef
		60 × 20		66.0a	37.2bc
		60 × 25		64.9ab	41.1b
	7/20	60 × 15	9/3 (46)	55.8d	23.8g–l
		60 × 20		54.5de	29.2ef
		60 × 25		51.7e	31.4de
	Mean			61.4	34.1
Hongeon	7/1	60 × 15	8/2 (33)	43.8f–i	20.7jkl
		60 × 20		43.2g–i	23.5h–l
		60 × 25		51.7e	28.1e–h
	7/10	60 × 15	8/11 (33)	46.7f.	20.4kl
		60 × 20		45.4fg	22.7i–l
		60 × 25		44.8fgh	25.8f–i
	7/20	60 × 15	8/22 (34)	42.1hij	18.7l
		60 × 20		41.1ij	23.5h–l
		60 × 25		39.4j	28.7e–h
	Mean			44.2	23.6
Arari	7/1	60 × 15	8/16 (47)	62.2bc	31.6de
		60 × 20		61.2c	35.2cd
		60 × 25		63.1abc	39.3bc
	7/10	60 × 15	8/24 (46)	63.1abc	27.2e–i
		60 × 20		62.8abc	35.6cd
		60 × 25		61.1c	37.5bc
	7/20	60 × 15	9/3 (46)	54.6de	21.9i–l
		60 × 20		53.8de	25.6f–k
		60 × 25		52.0e	29.0efg
	Mean			59.3	31.4
Varieties				**	**
Seeding date				**	**
Sowing density				**	**
Varieties × sowing date				**	**
Varieties × sowing density				NS	NS
Sowing date × sowing density				NS	NS
Block				NS	NS
LSD				2.74	4.64

*NS* non-significant.

ANOVA’s test; **: significant at P < 0.01.

^z^Days from seeding to flowering.

**Table 10:** Yield characteristics of small red bean according to sowing date and spacing.

Variety	Sowing date (mm/dd)	Sowing density (cm)	Number of seeds/pod	100 seeds weight (g)	Seed yield (kg/10a)
Chungju	7/1	60 × 15	8.26gh	14.03l	261.0abc
		60 × 20	8.23gh	14.16jkl	243.3a–f
		60 × 25	8.20hij	14.23i–l	235.0b–f
	7/10	60 × 15	8.33e–h	15.10d–g	275.0a
		60 × 20	8.40d–h	15.30de	270.0a
		60 × 25	8.20hij	14.73e–k	224.3d–h
	7/20	60 × 15	8.20hij	15.96bc	231.3b–g
		60 × 20	7.90j	16.00bc	210.6f–j
		60 × 25	7.86j	16.06bc	188.0j–m
	Mean		8.18	15.1	237.6
Hongeon	7/1	60 × 15	8.70a–e	14.36h–l	222.6e–h
		60 × 20	8.86ab	14.43h–l	201.3g–l
		60 × 25	8.76abc	14.13kj	189.6i–l
	7/10	60 × 15	8.66a–f	14.86e–h	200.6h–l
		60 × 20	8.33e–h	14.50g–l	159.6m
		60 × 25	8.40d–h	14.56f–k	167.0lm
	7/20	60 × 15	8.60a–g	15.66cd	197.3h–l
		60 × 20	8.56b–h	15.20de	174.3klm
		60 × 25	8.70a–e	15.33de	168.0lm
	Mean		8.62	14.8	186.7
Arari	7/1	60 × 15	8.83abc	14.83e–i	257.6a–d
		60 × 20	8.93a	15.06efg	228.3c–h
		60 × 25	8.73a–d	14.76e–j	196.6h–l
	7/10	60 × 15	8.56b–h	15.16def	264.0ab
		60 × 20	8.60a–g	14.26h–l	246.0a–e
		60 × 25	8.50c–h	14.73e–k	207.3g–k
	7/20	60 × 15	8.33e–h	16.70a	226.0d–h
		60 × 20	8.33e–h	16.30ab	200.6g–l
		60 × 25	8.30fgh	16.06bc	179.6j–m
	Mean		8.57	15.4	222.9
Varieties			**	**	**
Sowing date			**	**	**
Sowing density			NS	*	**
Varieties × sowing date			**	**	**
Varieties × sowing density			NS	NS	NS
Seeding date × sowing density			NS	NS	NS
Block			NS	NS	NS
LSD			NS	NS	*

All data generated or analysed during this study are included in this published article.

*NS* non-significant.

ANOVA’s test; *, **: significant at P < 0.05, 0.01.

The average stem lengths of ‘Chungju’, ‘Hongeon’, and ‘Arari’ were 61.4 cm, 44.2 cm, and 59.3 cm, respectively. The stem lengths were significantly different between sowing dates and sowing density (Table 9), with July 10 and 20 producing the longest and shortest lengths, respectively. Moreover, the stem length was longest at 60 × 15 cm and shortest at 60 × 25 cm. ‘Chungju’ stem length was longest (66.0 cm) at 60 × 15 cm on July 1, ‘Hongeon’ (51.7 cm) at 60 × 25 cm on July 1, and ‘Arari’ (63.1 cm) at 60 × 15 cm on July 10.

However, ‘Chungju’, ‘Hongeon’, and ‘Arari’ stem lengths were shortest (51.7, 39.4, and 52.0 cm, respectively), at 60 × 25 cm on July 20. The stem length increase with higher planting density could be attributed to the competition for nutrients between plants. Similarly, the narrower the interval between plants, the longer the soybean stems, from late sowing with second cultivation after wheat harvesting^66^. Other studies also reported similar results, where the culm length decreased with the delayed sowing date of the small red bean^62,67^.

The average number of pods per plant in ‘Chungju’, ‘Hongeon’, and ‘Arari’ were 34.1, 23.6, and 31.4, respectively. The number of pods was significantly different depending on the cultivation method, such as sowing dates and sowing density (Table 9), highest on July 1, and lowest on July 20. The pods were also highest at 60 × 25 cm spacing, while lowest at 60 × 15 cm. The number of pods was highest at 60 × 25 cm, in ‘Chungju’ (46.1 pods) on July 1, Hongeon (28.7 pods) on July 20, and ‘Arari’ (39.3 pods) on July 1.

Therefore, the number of pods per plant increases with decreased sowing density. Furthermore, the number of pods per plant in ‘Chungju’, ‘Hongeon’, and ‘Arari’ were lowest (23.8, 18.7, and 21.9, respectively) at 60 × 15 cm sowed on July 20, indicating that the number of pods increases with increased branches due to the long period of vegetative growth and the wider density between individual plants. Similarly, the number of seeds per plant in soybean increases with early sowing date and broader sowing density^68^.

The average number of seeds per pod was 8.18, 8.62, and 8.57 in ‘Chungju’, ‘Hongeon’ and ‘Arari’, respectively, and showed significant difference with sowing dates (Table 10). However, sowing density did not affect the number of seeds per pod. Overall, the average number of seeds per pod was highest (8.61) on July 1 and the lowest (8.31) on July 20. The total number of seeds per pod was about 8, similar to Hong et al.indicating that the number of seeds per pod is similar regardless of the sowing date^67^. However, the average number of seeds per pod of ‘Chungju’, ‘Hongeon’ and ‘Arari’ was 6.0, 6.5, and 6.8, respectively, as presented in NICS^69^, the average score of the national adaptation experiment conducted for over three years. In this study, the number of seeds per pod was about 2 seeds higher than the national average.

The 100-grain weight was 15.1, 14.8, and 15.4 g for ‘Chungju’, ‘Hongan’, and ‘Arari’, respectively, significantly different at various sowing dates (Table 10). The 100-grain weight was highest on July 20 and least on July 1. The weight was also highest at 60 × 15 cm and least at 60 × 25 cm. The 100-grain weight was highest in ‘Arari’ (16.7 g) at 60 × 15 cm on July 20 and least in ‘Chungju’ at 60 × 15 cm on July 1. The average 100-grain weight was 14.0 g, and it increased with delayed sowing. Similarly, Kang et al.indicated that the weight of the seeds increases with delayed sowing of the small red bean^70^.

The ‘Chungju’, ‘Hongeon’, and ‘Arari’ yields were 237.6, 186.7, and 222.9 kg per 10a, respectively, and showed a significant difference between sowing date and sowing density. The yield was highest on July 1 and lowest on July 20. Besides, the yield was highest at 60 × 15 cm and least at 60 × 25 cm. The yield was highest at 60 × 15 cm, in ‘Chungju’ on July 10, ‘Arari’ on July 10, and ‘Hongeon’ on July 1 (Table 10). Similarly, the soybean yield was higher with increased sowing density^63^. Furthermore, the yield was lowest in ‘Hongeon’ at 60 × 20 cm on July 10, in ‘Arari’ at 60 × 25 cm on July 20, and in ‘Chungju’ on July 20 at 60 × 25 cm, (159.6, 179.6, and 188.0 kg per 10a, respectively). ‘Chungju’ and ‘Arari’ yield were higher on July 1 and July 10 than July 20 sowing. However, the ‘Hongeon’ yield was similar on different sowing dates.

Therefore, a high yield was obtained at an earlier sowing date and shorter sowing density. Besides, the three varieties had good yield even when sowed on July 20. For instance, ‘Hongeon’ sowing was delayed due to the bad weather conditions, such as rainfall after harvesting wheat, increasing the sowing period compared to ‘Chungju’ or ‘Arari’, thus high yield.

## Conclusion

The study results demonstrated that ‘Goso’ (wheat) and ‘Chungju’ (small red bean) are the best varieties for Hodugwaja production. In this study, ‘Goso’ and ‘Chungju’ had high yields, good growth characteristics for cookies, and superior characteristics of bean sediment. Our findings reveal that in a double cropping system, ‘Goso’ sown on October 26 with additional nitrogen fertilization at 200% of the standard (N3), and ‘Chungju’ sown on July 10 at a sowing density of 60 × 15 cm in a high ridge (25 cm) cultivation method, produces the highest yields. However, more studies are necessary for the standard fertilization level of nitrogen in wheat cultivation.

## Data Availability

All data generated or analyzed during this study are included in this published article. The original datasets generated during and/or analyzed during the current study are available from the corresponding author on reasonable request.
